# Electrospun Nanofibers for Biomedical, Sensing, and Energy Harvesting Functions

**DOI:** 10.3390/polym15214253

**Published:** 2023-10-29

**Authors:** Didem Demir, Nimet Bolgen, Ashok Vaseashta

**Affiliations:** 1 Chemistry and Chemical Process Technologies Department, Mersin Tarsus Organized Industrial Zone Technical Sciences Vocational School, Tarsus University, Mersin 33100, Türkiye; didemdemir@tarsus.edu.tr; 2 Chemical Engineering Department, Faculty of Engineering, Mersin University, Mersin 33110, Türkiye; nimetbolgen@yahoo.com; 3 Applied Research, International Clean Water Institute, Manassas, VA 20110, USA; 4 Institute of Biomedical Engineering and Nanotechnologies, Riga Technical University, LV 1048 Riga, Latvia

**Keywords:** electrospun nanofibers, biomedical technologies, sensors, e-textile, energy harvesting

## Abstract

The process of electrospinning is over a century old, yet novel material and method achievements, and later the addition of nanomaterials in polymeric solutions, have spurred a significant increase in research innovations with several unique applications. Significant improvements have been achieved in the development of electrospun nanofibrous matrices, which include tailoring compositions of polymers with active agents, surface functionalization with nanoparticles, and encapsulation of functional materials within the nanofibers. Recently, sequentially combining fabrication of nanofibers with 3D printing was reported by our group and the synergistic process offers fiber membrane functionalities having the mechanical strength offered by 3D printed scaffolds. Recent developments in electrospun nanofibers are enumerated here with special emphasis on biomedical technologies, chemical and biological sensing, and energy harvesting aspects in the context of e-textile and tactile sensing. Energy harvesting offers significant advantages in many applications, such as biomedical technologies and critical infrastructure protection by using the concept of finite state machines and edge computing. Many other uses of devices using electrospun nanofibers, either as standalone or conjoined with 3D printed materials, are envisaged. The focus of this review is to highlight selected novel applications in biomedical technologies, chem.-bio sensing, and broadly in energy harvesting for use in internet of things (IoT) devices. The article concludes with a brief projection of the future direction of electrospun nanofibers, limitations, and how synergetic combination of the two processes will open pathways for future discoveries.

## 1. Introduction to Electrospun Nanofibers

One-dimensional nanomaterials in the form of nanofibers have a wide-ranging spectrum of research and commercial applications, due to their exceptional physicochemical properties and characteristics. Electrospinning is a relatively simple, yet versatile and promising method for the production of nanofibrous materials for a wide variety of applications [1,2,3,4,5,6,7,8]. A typical electrospinning apparatus consists of a high-voltage power supply to generate an electrical field, a syringe pump with speed and volume control to provide the polymer or polymer blend flow rate, and a collector plate on which the nanofibers to be formed are deposited. In this method, which is a conventional electrospinning setup, the following processes proceed, respectively: (i) polymer liquid is fed by a syringe pump at a given flow rate; (ii) under the strong electric field generated, the polymer liquid fed as a spherical droplet gradually transforms into a cone (referred to as a Taylor cone) and a liquid jet is formed; (iii) the charged polymer jet extends and tapers along a straight line and solidifies and accumulates on a grounded collector plate to produce a solid fibrous mat. The shape of the cone, in the presence of process parameters such as viscosity; conservation of mass, charge, momentum; electric field; surface tension; air drag; and Coulombic and gravitational forces, has been modeled by several investigators [9]. In addition, the morphology of fibers may vary depending on the process parameters (polymer feed rate, applied voltage, distance between the needle and collector, needle diameter, and collector type), environmental conditions (temperature, humidity, atmospheric flow, and pressure), and the properties of the solution (viscosity, conductivity, polymer concentration, surface tension, elasticity, and solvent volatility) [10].

**Figure 1:**
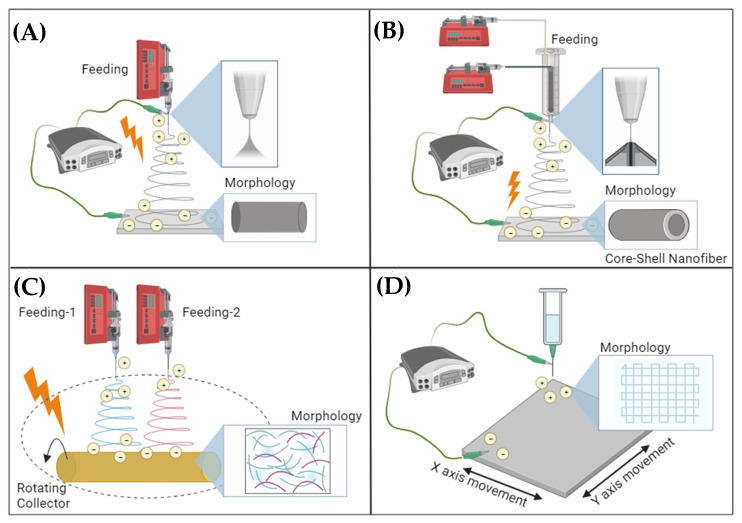
Electrospinning configurations (**A**): basic electrospinning apparatus, (**B**): co-axial electrospinning, (**C**): dual jet electrospinning, and (**D**): near-field.

These nanofibers with cross-sectional diameters ranging from tens to hundreds of nanometers possess high surface energy due to a large surface area. Furthermore, they can form networks of highly porous mesh with remarkable interconnectivity between their pores, making them attractive for a multitude of innovative applications. The materials for nanofibers include natural polymers, synthetic polymers, carbon-based, semiconductors, ceramers, and composites. A few basic configurations of electrospinning apparatus are shown in Figure 1A–D, and many other configurations such as co-axial, multi-axial, and multiple jets; and melt electrospinning [11], solution electrospinning [12], solvent-free electrospinning [13], near-field electrospinning [14], and conjugated electrospinning [15] are reported in the literature for applications-specific fiber production.

Electrospun nanofibers exhibit exceptional and unique properties including high extensibility and high mechanical strength. Electrospinning technology has gained enormous interest in recent years both as a research topic and in futuristic industrial fields. Tremendous efforts have been devoted to the development of electrospinning process setups, strategies for successful electrospinning, characterization of electrospun nanofibers, and exploring novel applications of electrospun nanofibers in different fields. Significant improvement has been achieved in the development of electrospun nanofibrous matrices, which includes tailoring compositions of polymers and active agents, surface functionalization with nanoparticles, and encapsulation of functional materials within the nanofibers. Electrospun nanofibrous materials have attracted significant attention, due to the flexibility of electrospun nanofibers, providing suitable mechanical property requirements and high surface area for a wide variety of applications. The unique properties of electrospun materials, including high surface-to-volume ratio, flexibility, high mechanical strength, high porosity, and adjustable nanofiber and pore size distribution, render them potential candidates in a wide range of applications in medical and engineering fields. Electrospinning is becoming more efficient and more specialized in order to produce particular fiber types with variable diameter and morphology, tunable alignment, patterned, and 3D structure. By controlling electrospinning polymer parameters, additives, and production strategies, it is possible to control nanofibrous structures, with specific properties for applications in tissue engineering and regenerative medicine, textiles, water treatment, sensors, and energy fields.

## 2. Electrospun Nanofibers for Biomedical Applications

**Figure 2:**
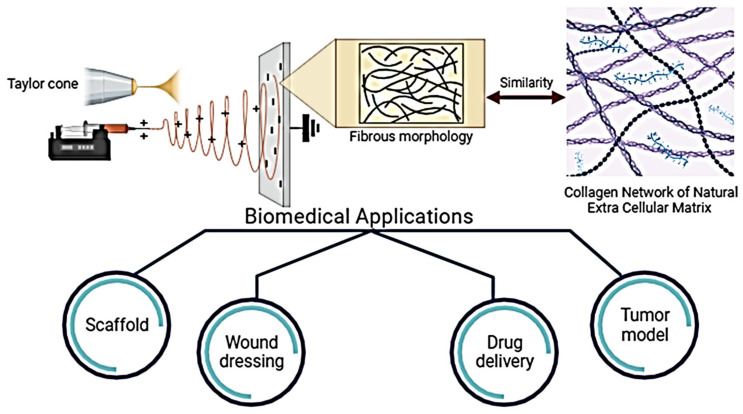
Biomedical applications of micro/nanofibers produced by electrospinning: scaffolds for tissue regeneration, wound dressings, drug delivery systems, and 3D tumor models.

Biomaterials in the form of coatings, particles, spheres, fibers, thin films, foams, and gels can be designed using many different conventional or modified techniques developed for product manufacturing in the biomedical field. The main feature in the development of biomaterials for biomedical applications is biocompatibility, and according to this feature, Bonferoni et al. divided biomaterials into four categories in their study. In this case, first-generation biomaterials were characterized as bioinert, second-generation biomaterials as absorbable or bioactive, third-generation materials as both absorbable and bioactive, and fourth-generation biomimetic materials [16]. As the fourth generation, including today, electrospun nanofibers have attracted great scientific interest, especially the biomimicking of the hierarchical architecture and fibrous structure of the native extracellular matrix (ECM) [17,18]. In addition, they have a large surface/volume ratio, are lightweight, have a porous morphology, and have the potential to load bioactive molecules into the fibers. Electrospun fibers arranged randomly and aligned can be fabricated in morphologies such as rod, core–shell, hollow, and porous depending on the process variables, solution parameters, and environmental conditions [18,19,20]. Natural or synthetic polymers are used alone or in combination in the production of fibers. The most commonly used polymers in fiber production for biomedical applications can be listed as poly(ε-caprolactone) (PCL) [21], gelatin [22], chitosan [23], silk fibroin [24], collagen [25], poly(lactic acid) (PLA) [26], and poly(lactic-co-glycolic acid) (PLGA) [27]. In addition, the fibers produced with active components such as nanoparticles, drugs, essential oils, and plant extracts included in the polymer structure can provide functional properties such as antibacterial, antiviral, antioxidant, and wound healing activity. Among the most used functional substances are essential oils and metal nanoparticles (silver, zinc, iron, titanium, etc.). After a general introduction to the use and design of electrospun fibers for biomedical applications, in the following part of our study, the customization of electrospun fibers as scaffolds for tissue regeneration, wound dressings, drug delivery systems, and 3D tumor models (Figure 2), studies, and current approaches for biomedical applications are mentioned.

### 2.1. Scaffolds

Scaffolds are biomaterials, as one of the fundamental components of tissue engineering, developed for the repair/restructuring of lost/damaged tissues as a result of any accident or disease, congenital anomalies, or trauma [28]. In the normal process, living tissues and organs can self-renew, but are quite limited when faced with critical-size defects. In this case, temporary support materials are needed until new tissue formation is achieved in the damaged area to ensure healing. For this purpose, scaffolds produced by processing monomer/polymers under laboratory conditions are artificial structures that imitate the native ECM architecture. Fibers at the nano- and microscale manufactured using electrospinning are one of the most important scaffold types used for this imitation by providing a micro-/nano-level cell-friendly environment to guide cell attachment, proliferation, differentiation, and migration. Up to now, scaffolds in electrospun mesh structures have been studied for a variety of tissues including bone, cartilage, cardiovascular, skin, nerve, and periodontal tissues [29,30,31]. In studies on the use of electrospun fiber structures as scaffolds, it is observed that researchers are focused on composite fibers that gain multi-functional properties by adding different additives, not polymers alone. In Table 1, current studies on this subject are summarized together with the biopolymer used in fiber production, the additive added to functionalize it, fiber properties, and application area. As seen in the studies, the combination of electrospun fibers and bioactive particles is a successful strategy for fabricating electrospun scaffolds for different tissue engineering applications. The nanoparticle–fiber combinations are formed by three different strategies. These are adding nanoparticles directly to the polymer solution before electrospinning, feeding them from a separate line during fiber production by coaxial electrospinning, or synthesizing nanoparticles on the mats after the fibrous membrane is produced [32]. Although nanoparticle–nanofiber combinations created using all these strategies exhibit highly functional properties, there are still questions about the toxicity of nanoparticles due to their accumulation in living organisms. For instance, metal-based nanoparticles have been linked to increased oxidative stress and have the potential to leak into the cell nucleus, and protein-based nanoparticles have been observed to have hepatotoxicity and nephrotoxicity as side effects [33]. Therefore, toxicological studies are also needed to provide a more detailed analysis of nanoparticles used in biomedical applications.

**Figure 3:**
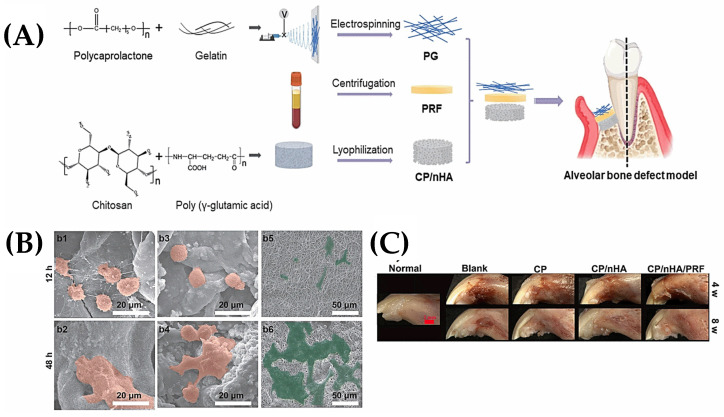
The scaffold design in multiple layers for guided bone regeneration. (**A**) Fabrication of each layer. (**B**) Cell behavior on scaffold surfaces ((b1–b2: CP, orange; b3–b4 CP/nHA, orange; b5–b6: PG, green). (**C**) Alveolar bone repair in different scaffolds at 4 and 8 weeks with optical images of retrieved specimens. Copyright @Elsevier, reproduced with permission from Elsevier [50].

Apart from the addition of bioactive compounds, electrospun fibers are also used in the design of multi-part scaffolds by producing gradient layers [44,45,46] and combining different structures including hydrogels [47] and 3D printed patterns [48,49]. The integration of electrospun nanofibers designed for tissue regeneration or new tissue formation processes with 3D systems is particularly important for the mimicry of hierarchical tissues. Recent studies on this subject will enable the design of new biomimetic scaffolds in the coming years. For instance, in a recent study, three-layered scaffolds consisting of hydrogel, platelet-rich fibrin, and nanofibers were designed by Zhang et al. for guided tissue regeneration and guided bone regeneration applications. PCL and gelatin (PG) were used to produce electrospun nanofibers, and in vitro and in vivo experiments were performed with the hybrid scaffold used by placing them on top of each other according to the design given in Figure 3A. After 48 h, cells showed multilateral elongation and expanded polygonal filopodia on the scaffold surfaces. In particular, they showed significant spread, covering most of the nanofiber surface (Figure 3B). In the evaluation of alveolar bone regeneration experiments, PG nanofibers were applied to the soft tissue surface as the outermost layer of the composite scaffolds (CP and CP/nHA, Figure 3C). The nanofibers of 150 μm thickness and approximately 700 nm fiber sizes served as a barrier against connective tissue infiltration, providing a more suitable area for bone regeneration [50].

### 2.2. Wound Dressings

A wound is defined as a disruption of skin integrity and normal functioning as a result of various diseases, traumas, burns, or illnesses. Especially in the case of chronic and deep wounds, it is important to cover and protect the wound with dressings and rebuild a temporary body barrier. Current wound dressings used for wound healing are cost-effective but have limited functionality to meet the needs of the existing market. For example, they cannot accommodate complex wound healing environments, so healing is slow, or they can adhere to the wound surface, which can cause additional damage and pain when they are replaced [51]. To overcome the handicaps of existing materials, scientists have started to design new-generation materials with a tissue engineering and materials technology approach. These materials include hydrogels [52], sponges [53], hydrocolloids [54], solvent casting films [55], and electrospun nanofibers [56,57]. Among them, fibrous membranes produced using the electrospinning technique are widely preferred to be used as wound dressing material. The main beneficial properties of electrospun nanofibers for wound moistening, cell growth, cell respiration, and skin regeneration are their larger functional surface, high porosity, good mechanical properties, and excellent biocompatibility. In addition, nanofibers can meet the different needs of wound treatment by encapsulating active agents (drugs, growth factors, living cells, cytokines, or inhibitors) or by incorporating them into their structural design that will accelerate wound healing. This is because during the inflammatory phase of wound healing, immune cells secrete pro-inflammatory cytokines, causing inflammatory cells to produce reactive oxygen species (ROS). Excessive ROS accumulation may increase the inflammatory response by damaging the antioxidant defense system. Thus, wound healing is delayed and scar formation occurs [58]. To prevent this, natural products of plants with antioxidant, antiviral, and antibacterial properties can be added to the fiber structures [59]. The most preferred polymers for the production of electrospun fibers as wound dressings are gelatin, chitosan, silk fibroin, hyaluronic acid, cellulose, alginate, poly(vinyl pyrrolidone) (PVP), PCL, and poly(vinyl alcohol) (PVA). In Table 2, current studies with wound dressing materials prepared based on electrospun fibers are compiled.

In addition to active ingredients that exhibit antibacterial properties, extracts of many different plant species have been used in the design of wound dressing materials. In the studies conducted in this context, the changes caused by herbal additives in the material are examined by revealing the physicochemical properties. The issues that need to be emphasized and developed are the diffusion of these bioactive components into the wound, release behavior, and release modeling. There is also a need to improve the effects of such additives on the mechanical properties of the material.

### 2.3. Tumor Model

Cancer is a difficult disease that affects millions of people worldwide, is a source of high stress, and has become the leading cause of death. Therefore, it is of great importance to investigate the formation, invasion, and metastasis of the malignant tumors that cause cancer. One of the most important steps taken in this context is in vitro cancer models that mimic the microenvironment where cancerous cells are found and a tumor develops [70]. In vitro cancer models are prepared in the same way as scaffolds that host cells for new tissue formation, with the tissue engineering approach. Electrospun scaffolds are used in this context as one of the most suitable structures that can mimic the micro-framework of a native tumor ECM and have high versatility by loading biochemical stimuli, including growth factors, adhesion molecules, and drugs [71]. To best mimic the fibrillar structure of a native ECM, it is desirable to include both nano- and sub-microfibers within the structure. As an example, in a study conducted by Luo et al., combined electrospinning and modified in situ biosynthesis method was used to fabricate a tumor model consisting of bacterial cellulose nanofibers and electrospun cellulose acetate sub-microfibers [72]. 

In addition, the topography of fibers plays a significant role in mediating neoplastic invasion and metastasis. In a study by Ali et al., they evaluated the scaffolds prepared with PCL and PLA polymers to create a 3D model of colorectal adenocarcinoma. By changing the polymer type and fiber alignment, changes occurred in the mechanical properties of the scaffold, hydrophobic properties, pore size, and porosity. Although Caco-2 cells managed to colonize in each tumor model, an opposite trend of cell metabolic activity occurred in PLA and PCL scaffolds as a function of fiber alignment, increasing in PLA and decreasing in PCL scaffolds [73]. 

Electrospun fibrous structures prepared based on different natural and synthetic polymers have been mostly used in breast cancer in vitro modeling studies. Studies conducted in this context include chitosan/PEO [70], collagen-based [74], cellulose acetate/silk fibroin [75], and PLGA/PEO [76] nanofibrous composites for 3D in vitro culture models. Apart from breast cancer modeling, electrospun fibers have also been evaluated for pancreatic cancer, colorectal cancer, prostate cancer, Ewing sarcoma, glioblastoma, melanoma, lung cancer, and bladder cancer [71].

### 2.4. Drug Delivery Systems

Drug delivery systems describe technologies and materials developed to deliver a therapeutically effective amount of drug to the damaged/diseased area. Electrospun nanofibers have emerged as promising materials for the construction of nanoscale drug delivery platforms because of their large surface-to-volume ratio, high drug loading capacity of up to 60%, nearly 100% encapsulation efficiency, ability to modulate drug release, and controllable surface modification [77,78]. The area/volume ratio combined with the current capability to produce biocompatible and biodegradable fibers reproducibly offers tremendous promise for diverse biomedical applications from tissue-engineering-targeted vaccine delivery and non-thrombogenic materials for blood-contacting applications. Biodegradable polymers are highly desirable for long-term drug delivery applications because they disintegrate in the body into biologically inert and compatible molecules. By combining drugs into a biodegradable polymer matrix, dosage forms of various shapes and sizes can gradually distribute the intended drug over prolonged periods of time. Secondary surgical procedures common after the completion of dosing regimens will not be needed as the remaining polymer dosage naturally disintegrates as well. Thus, biodegradable polymers offer a novel approach to developing simple and convenient sustained-release drug delivery systems. Many biodegradable polymer chemistries have been proposed for such applications; however, the most common and successful are polyesters first investigated as degradable sutures. These polymers include poly(glycolide), poly(D, L-lactide), and their related copolymers PLGA. Current commercial products based on these materials include Decapeptyl^®^, Lupron Depot^®^, and Sandostatin LAR^®^.

Although the word “drug” is used to express release here, the loading of synthetic, natural, and biologically active substances into electrospun nanofibers and examining the release of these substances are also considered in this category. Therefore, the release of active substances ranges from various drugs such as antibiotics, anti-inflammatory, antimicrobial, cardiovascular, antihistamine, gastrointestinal, palliative, and contraceptive to macromolecules such as proteins and DNA [79]. Polymeric nanofibers and a drug can be brought together in various ways and in different physical states. These states are, as summarized by Wang et al. [80]: (i) homogeneous dispersion of the drug throughout the polymer fiber, (ii) crystalline dispersion of the drug on the surface of the polymer fiber, and (iii) encapsulation of the drug by polymer fiber. In Table 3, recent studies reported in the literature are summarized in terms of new strategies used, the polymer selected in fiber production, the active substance to be released, and the application area. Electrospun nanofibers are promising as targeted drug carriers for in situ delivery of cancer drugs, especially for chemotherapy. 

Despite the great advantages of drug-loaded fibrous materials produced on the basis of electrospinning, the number of clinical studies reported in the literature is not yet sufficient. In electrospinning, toxic residues of the solvents chosen to dissolve the polymers may be trapped in the fibers and released from the fiber along with the drug during the release period. For this reason, there is a need to focus on solvent selection studies and develop new approaches that utilize the use of more environmentally friendly and green solvents such as water instead of aggressive and toxic ones [91].

## 3. Chemical Sensors and Biosensors 

The electrospinning process is a unique method to produce nanofibers of varying porosity and thus highly effective areas with large surface activation energy for sensing applications. Several combinations of synthetic and natural polymers; polymer composites; and polymers impregnated with nanoparticles, carbon nanotubes, ceramers, and other compounds have been synthesized for specific applications [2]. Depending upon the additives, the characteristics of the synthesized nanofibers can be custom-tailored for very specific applications. In addition, nanoparticles can be functionalized for specific receptors [5,8]; thus the nanofibers synthesized with such functionalized nanoparticles can be designed to capture specific targets, thus producing chemical and biological sensors as devices that can detect biological information about the human body to determine whether there is a possibility of disease [92,93]. According to the sensing principle of the biosensor device with electrosensing technology, it can be used as piezoelectric, electrochemical, thermal, and optical biosensors [94,95,96]. 

Electrochemical biosensors are the best candidates for measuring specific biomarker concentrations in body fluids [97]. To increase the effectiveness of these biosensors, DNA, enzymes, carbon-based materials, and nanoparticles are usually immobilized on the electrode surface of the sensors [98]. The enzymes had high sensitivity, selectivity, and catalytic efficiency, thus having a significant impact on the performance of the sensors [99]. The most common study on sensors produced with electrospinning nanofibers is on the production of enzyme electrochemical sensors.

**Figure 4:**
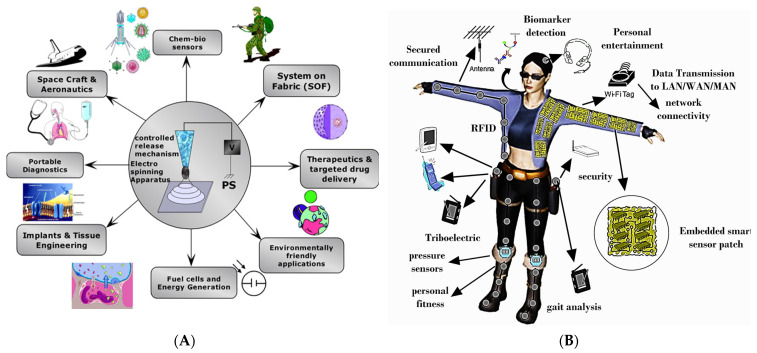
(**A**) Targeted and potential applications of polymers/ceramers-based electrospun fibers and (**B**) conceptual architectural layers of SoF in wearable layers (produced with permission) [102].

The onset of mobile and pervasive computing; wireless networks; network appliances; flexible substrates; and e-textile and tactile sensors is enabling the development of systems on textiles or systems on fibers (SoFs). The ability to disperse ceramics, metal oxides, nanoparticles, and carbon nanotubes in polymer solutions and develop fibers, thereafter employing electrospinning, has produced new pathways for functional fibers, mentioned here as SoFs—that have electronics and interconnections woven into them. Such systems demonstrate incredible potential for the development of portable functional devices such as fibers embedded with data communications systems, nanogenerators for energy harvesting, health and biomarkers monitoring, personal fitness, defense systems, mobile computing, targeted and time-released delivery of vaccines, protective armor, chemical-biological sensors, radio frequency identification (RFID) devices, Global Positioning System (GPS), and interactive geographical information systems (GIS). Additive processing technology offers the possibility for the development of systems with physical flexibility and size unachievable with current and conventional advanced manufacturing technology [100,101]. Recent advances in finite state machines (FSMs), machine core learning (MCL), and edge computing architecture have led to the introduction of distributed network systems of IoTs, capable of sensing and detection in a remote and distributed environment. Additionally, the use of e-textiles, tactile sensors with computing systems and signal transmission capabilities, provides warfighters with situational awareness in an austere environment. The vision of SoF sketches a scenario that enables context-aware functionality, a data communication interface, parallel detection of chem.-bio sensors, and invaluable real-time information for soldiers in combat theatres to execute critical decisions in a timely manner. Figure 4A outlines a comprehensive overview of some of the targeted applications of our current and ongoing programs and Figure 4B shows a conceptual architectural layer of SoF in wearable layers adopted for civilians and soldiers in combat theatres. Some of the functionalities include energy harvesting aspects, which are further described in the following section.

## 4. Energy Harvesting and Tactile Sensing

Due to the highly interconnected nature of our environment, there has been a growing need for ubiquitous sensing for enhanced situational awareness. One of the primary needs for ubiquitous sensing is to power/activate these devices in a standalone mode since a distributed power grid may not have the power option in the field for mobile, remote, and free-standing applications. Several sensors based on the piezoelectric effect offer the advantage of self-powered devices, however, have so far been limited to bulky and inflexible sensors. Recent interest in nanofibers doped with poly(vinylidene fluoride) (PVDF) and lead zirconate titanate (PZT) and PZT doped with different lanthanides inorganic fillers [103] have demonstrated the feasibility of providing energy harvesting from the environment as capacitive, piezoresistive, and tactile sensors based on the piezoelectric effect.

**Figure 5:**
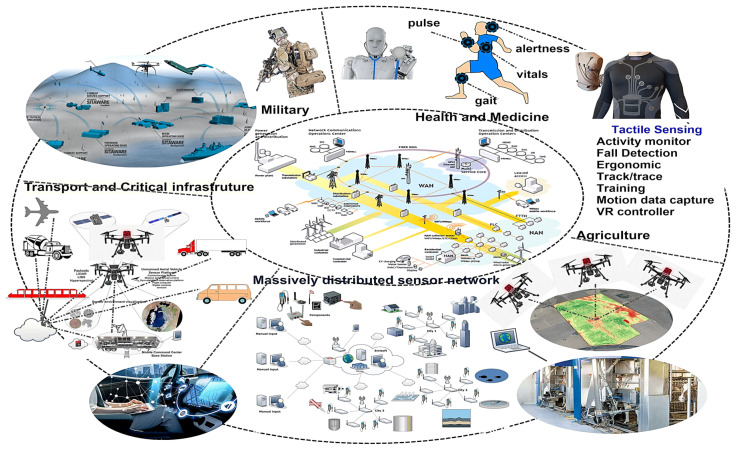
The application spectrum of the potential application of energy harvesting aspects of tactile and flexible sensors.

PVDF is ideal as a sensing element for tactile sensing due to its low cost, flexibility, and biocompatibility. PVDF also possesses good thermal stability, low permittivity, and wide frequency response, and has excellent thin-film-forming properties [104]. PVDF has a carbon chain as its basic skeleton and is significantly more flexible than single crystals and ceramics. This increased flexibility allows it to withstand greater strain, making it more suitable for tactile sensing scenarios that involve substantial bending and twisting. The piezoelectric nature of PVDF is attributed to its phase structure and it has a piezoelectric nature due to its polar phase. This transformation step is a significant drawback of using PVDF as a functional element as the polar phase transition techniques such as high-temperature annealing, high voltage electric poling, and mechanical stretching are cost-intensive and can affect the device fabrication process [105]. Hence, different fillers are considered alternative methods that effectively induce piezoelectric performance by creating nucleation sites in the PVDF matrix, which induces a crystalline structure. Fillers such as lanthanide-doped PZT, barium titanate (BaTiO_3_), and trifloroethylelene (TrFE) have been experimented with to enhance performance figures of merit [103]. These efforts have intensified the development of new materials for smart sensors with high sensitivity and reliability. Tactile sensors, also known as touch sensors, play a crucial role in mimicking human-like tactile perception and have already found applications in the fields of robotics and medical devices. Our efforts have been to use these sensors for critical infrastructure monitoring, biomedical applications, smart agriculture, smart and connected city projects, and tactile sensing for robotics. An overview of these efforts is shown in Figure 5, which shows an application spectrum of the potential application of energy harvesting aspects of tactile and flexible sensors.

As biomedical sensors, PVDF-based electrospun fibers are highly suitable for piezoelectric sensor applications due to their high piezoelectric, pyroelectric, and ferroelectric properties with high mechanical strength, high thermal stability, superior flexibility, and excellent biocompatibility [106]. However, the figure of merit (FOM) of PVDF-based membranes remains quite low. In an effort to enhance the piezoelectric response, several experiments are in progress with significantly higher longitudinal and transverse piezoelectric strain coefficients (d_ij_), viz. lead zirconate titanate (PZT), lanthanum-substituted PZT, lithium niobate (LiNbO_3_), and lead magnesium niobate-lead titanate (PMN-PT). 

PVDF-based sensors/energy harvesters show promise for the early evaluation and prevention of cardiovascular diseases and respiratory disorders, as well as many pressure-related acute and chronic diseases [107]. In recent years, flexible electronics and integrable sensors for wearable human motion monitoring and tactile sensing applications have shown rapid progress [108]. As an example, Li et al. studied a one-step strategy for fabricating core/shell PVDF/dopamine nanofibers [107] for biomedical sensors by testing the produced sensor on human skin and in vivo in mice. The piezoelectric sensor provided an accurate reading of weak mechanical excitations caused by blood vibrations and was able to accurately and rapidly detect changes in diaphragmatic contraction and peripheral arterial walls in different physiological conditions. Limited flexibility of conventional materials restricts their applicability in tasks involving wide strain ranges. Sengupta et al. developed a wearable and sensitive carbon nanofiber/polydimethylsiloxane piezoresistive sensor realized by carbonizing electrospun polyacrylonitrile nanofibers and embedding in PDMS elastomeric thin films for tactile sensing applications [109]. The feasibility of applying sensors for wearable devices and to track knee joint movements was demonstrated. It was concluded that such devices in tandem with neuromorphic circuits could potentially recreate the sense of touch in robotic arms and restore somatosensory perception in amputees. In another study, Ramados et al. fabricated a pressure sensor by using the electrospinning method and attached the sensor to a commercially available robotic arm for robotic tactile sensing applications. They used a low-cost atactic polystyrene material to develop the pressure sensor and the fabricated sensor attached to the robotic arm to mimic tactile sensing, and the corresponding voltage output, 13V, was estimated to lift a 400 gr aluminum bar [110]. Other applications of such sensors have been studied for heart rate, sweating, sleep, blood sugar, blood pressure, body temperature, catheter placement, sitting posture adjustment, and hand/body tremor monitoring [111]. 

## 5. Conclusions, Recommendations, and Path Forward

Research on electrospun nanofibers is growing at a tremendous pace, especially due to the numerous advantages and benefits that nanofibers have to offer in various fields which include tissue engineering, wound dressing, drug delivery, biomedical engineering, energy harvesting, biosensors, and tactile sensing applications. The development in electrospinning technologies and combination of electrospinning with additive manufacturing techniques, such as 3D/4D printing, can lead to new strategic applications of this technology in the forthcoming years. Combining the electrospinning fabrication method with various types of materials including synthetic and natural polymers to fabricate tissue engineering scaffolds, biosensors, energy harvesting devices, and sensors could pave new paths and new research directions and industrial applications. Electrospun nanofibers with improved mechanical and functional properties can be manufactured by combining nanoparticles, active agents, and functional materials. However, although electrospinning technology has been greatly developed and received attention in the past two decades, there are still certain challenges with perfectly controlling the process parameters to produce nanofibrous materials with the same features consistently and improving the large-scale production speed of electrospinning for commercialization. More specifically, controlling porosity presents a challenge. Addition of materials to modify the fiber characteristics for desired applications presents a challenge as well. With the advent of finite state machine configuration, the use of nanogenerators in tactile sensors will play a major role in prosthetics and other functions that relate to monitoring body vitals remotely. Several applications in critical infrastructure are also possible and are currently being investigated. The application of electrospinning technology in the field of human health should be expanded to have a clear and deep understanding. The drug delivery systems produced by electrospinning to provide desired delivery profiles should focus on applying clinical trials to demonstrate effective treatments. Lastly, our efforts are towards a more sustainable future by taking into account life cycle of micro/nano particles [112] and plastics and also towards the use of bio-based polymers to reduce their environmental impact.

## Figures and Tables

**Table 1:** Current studies on fiber-based functional scaffolds produced by electrospinning.

Electrospun Polymer	Additives	Morphology	Application	Ref.
PCL	nanosilicate, laponit	randomly oriented filamentous architecture	periodontal regeneration	[34]
silk fibroin PCL	bioactive glass nanoparticles	double-layer scaffold with an oriented fiber topology	guided bone regeneration	[35]
PCL	graphdiyne nanoparticles	rough surface with a multilayer structure by rolling into the nerve guide conduits	peripheral nerve regeneration	[36]
PLGA	magnesium and zinc metallic particles	bi-layered membrane with both dense and porous structure	periodontal tissue regeneration	[37]
PEOT PBT	hydroxyapatite and mesoporous bioactive glasses	porous structure with random fiber orientation	bone tissue regeneration	[38]
PLLA-PCL	silk fibroin vitamin B5	double-layer sponge tube including both nanofiber and nano yarn	urethral regeneration	[39]
PCL	tropoelastin	wave-like fibers aligned in the stretch direction	musculoskeletal tissue mimicking	[40]
PLA	silk peptide cellulose nanofibrils Ag nanoparticles	randomly aligned, ultra-fine fibers covered with relatively smooth film	scaffolds for biomedical reconstruction	[41]
chitosan PEO	-	increased fiber diameters when using PEO	potential for cartilage repair applications	[42]
PHB-starch Chitosan-ECM	halloysite nanotubes	nanofibers in core–shell structure	potential for articular cartilage tissue regeneration	[43]

PEO: poly(ethylene oxide); PEOT: poly(ethylene oxide terephthalate); PBT: poly(butylene terephthalate); PLLA-PCL: poly(L-lactide-co-caprolactone); PHB: poly(hydroxyl butyrate); ECM: extracellular matrix.

**Table 2:** Current studies on fiber-based wound dressings produced by electrospinning.

Electrospun Polymer	Additive	Morphology	Acquired Property	Ref.
gelatin PEO ulvan	ulvan-stabilized silver nanoparticles	co-electrospun nanofibers	faster wound contraction during the early stages of the burn wound healing process	[60]
chitosan PVA	tannic acid	3D nanofiber sponge	antioxidant properties and antibacterial ability	[58]
PCL gelatin	strontium zinc silicon bioceramics	bioceramic-nanoparticle-loaded 2D fiber membrane	activation of hair follicle stem cells around the burn wound with ion release	[61]
PLA	beta-chitin whiskers, silver nanoparticles	bilayer membrane	hydrophilicity: moist environment, hydrophobicity: mechanical properties, antimicrobial activity: preventing skin wound infections	[62]
PAN	aloe vera extract and silver sulfadiazine tragacanth	sandwich electrospun nanofibers/tragacanth hydrogel composite	improved properties by adding hydrogel as a second layer to make a sandwich wound dressing	[63]
PCL chitosan	bromelain and silver nanoparticles	2D membrane with uniform and homogeneous fibers	additives improved the wound-healing process within one week compared to other groups	[64]
PCL PLA	*Capparis spinosa* L. zinc oxide nanoparticles	double-layer nanofiber membrane	additives enhanced the mechanical strength and good antibacterial effect	[65]
gelatin	*Hypericum perforatum* oil Vitamin A palmitate	active agents incorporated relatively uniform fibers	improved the properties of gelatin nanofiber	[66]
PU PCEC chitosan	TMP	2D membrane with homogeneous and smooth surface morphology	photosensitivity and bactericidal properties for cutaneous tissue healing	[67]
PCL chitosan pectin	-	2D membrane with uniform morphology	effective against a wide range of microbial organisms which aids in wound healing	[68]
zein pectin soy lecithin	vitamin C	continuous and smooth ribbon-like structure	promoting the healing and reducing inflammation in the created burn wound	[69]

PEO: poly(ethylene oxide), PAN: poly(acrylonitrile), PU: polyurethane, PCEC: poly(caprolactone)–poly(ethylene glycol)–poly(caprolactone), TMP: tetrakis (N-methyl pyridinium-4-yl) porphyrin tetratosylate salt.

**Table 3:** Current studies on fiber-based drug delivery systems produced by electrospinning.

Electrospun Polymer	Drug	Strategy	Application	Ref.
PLA	dicumarol	Before electrospinning, drug was linked to polymer by esterification reaction	Treatment of Peritendinous Adhesion	[81]
PAN	ibuprofen	Before electrospinning, ibuprofen was encapsulated in zinc oxide nanoparticles	Transdermal drug delivery carrier	[82]
PCL gelatin	curcumin	Before electrospinning, curcumin was encapsulated in zeolite Y nanoparticles	Postsurgical glioblastoma treatment	[83]
PDLLA	metronidazole	Co-electrospinning: the core was enriched with drug, while the sheath of the fiber consisted of polymer	Local drug delivery systems for the treatment of periodontitis	[84]
hyaluronic acid PHBV	icariin	Co-electrospinning: the core was made with icariin-loaded hyaluronic acid while the sheath was made from perovskite nanoparticle-loaded PHBV	Long-term osteogenesis-promoting application	[85]
PLGA	doxorubicin venetoclax	Directly adding to polymer solution before electrospinning	Synergistic inhibition of prostate cancer recurrence	[86]
pullulan PLGA	amoxicillin	Directly adding to polymer solution before electrospinning	Topical skin delivery applications	[87]
PLGA	doxorubicin paclitaxel	Sequential electrospinning of drug-containing polymer solutions	Inhibition of breast tumor in situ	[88]
chitosan PEO PCL	rosuvastatin	Co-electrospinning: the drug was electrospun together with the polymer solution to be found in the core of the fibers	Bone tissue engineering such as guided bone regeneration, bone fracture healing, and localized drug delivery to the damaged bone site	[89]
pluronic pectin keratin	mupirocin	Co-electrospinning: directly adding to the core polymer solution	Potential in wound healing applications	[90]

PDLLA: poly(L-lactide-co-D,L-lactide); PHBV: poly(3-hydroxybutyrate-co-3-hydroxyvalerate).

## Data Availability

Most information is contained in the manuscript. Additional data may be requested by the corresponding author and will be available upon reasonable request.
